# Optimizing the Synthesis of CO_2_-Responsive Polymers: A Kinetic Model Approach for Scaling Up

**DOI:** 10.3390/polym17081115

**Published:** 2025-04-20

**Authors:** Emil Pashayev, Prokopios Georgopanos

**Affiliations:** Helmholtz-Zentrum Geesthacht, Institute of Membrane Research, Max-Planck-Straße 1, 21502 Geesthacht, Germany; emil.pashayev@hereon.de

**Keywords:** CO_2_-responsive polymers, kinetic modeling of RAFT polymerization, upscaling

## Abstract

The kinetic model is a crucial tool for optimizing polymer synthesis protocols and facilitating the scaled-up production processes of the CO_2_-responsive polymer poly(N-[3-(dimethylamino)propyl]-acrylamide)-b-poly(methyl methacrylate)(PDMAPAm-*b*-PMMA), which is supposed to be implemented in direct air capture (DAC) technology. This study presents a simulation of the kinetic model developed for the Reversible Addition−Fragmentation Chain-Transfer (RAFT) polymerization of N-[3-(dimethylamino)propyl]-acrylamide (DMAPAm), alongside an investigation into the kinetics of this polymerization using the simulation as an analytical tool, as well as the application of the simulation for the upscaling of RAFT polymerization. Ultimately, the kinetic model was validated through two kinetic experiments, confirming its reliability. It was subsequently employed to optimize the synthesis recipe and to predict the properties of PDMAPAm homopolymers, thereby supporting the upscaling of PDMAPAm-*b*-PMMA diblock copolymer synthesis. In the end, the preliminary results of the CO_2_-responsiveness of the diblock copolymer were determined with a simple experiment.

## 1. Introduction

Carbon capture is a key area of research that is increasingly considered for application as a technology to mitigate CO_2_ emissions [1,2,3,4]. In a recent approach, carbon dioxide present in the atmosphere, even at relatively low concentrations, can be captured using a novel approach known as direct air capture (DAC) [2,3,5]. In DAC systems, it is essential to establish a stronger interaction between CO_2_ molecules and the solid adsorbent [3,5], and diblock copolymers can be considered adsorbents for DAC because of their capacity to form films with high porosity [6,7,8] and can also be scalable [9,10,11,12]. Diblock copolymers that incorporate amine functional groups are particularly effective at capturing CO_2_ due to the strong attraction that certain chemical groups to for carbon dioxide. A range of acrylate polymers, including poly(2-(dimethylamino)ethyl methacrylate) (PDMAEMA) [13,14], poly(N,N-dimethylallylamine) (PDMAAm) [15,16], poly(2-aminoethyl methacrylate) (PAEM) [17,18], and poly(N-[3-(dimethylamino)propyl]-acrylamide) (PDMAPAm) [19], which possess amine groups (responsive to CO_2_), can act as the foundational block for subsequent chain growth into diblock or triblock copolymers.

Controlled radical polymerization (CRP) is one of the most effective techniques for synthesizing well-defined block copolymers, because this polymerization technique enables the precise targeting of molecular weights and block ratios [20,21,22,23]. To date, three primary techniques of CRP have been explored and documented in the literature: atom transfer radical polymerization (ATRP), nitroxide-mediated polymerization (NMP), and Reversible Addition–Fragmentation Chain-Transfer Polymerization (RAFT) [24,25,26]. These established polymerization techniques allow for effective control over the reaction rate, dispersity, and molecular weight distribution (MWD) of the resulting polymer [27,28,29,30].

Compared to ATRP and NMP, RAFT-mediated polymerization offers the versatility to work with a wide variety of monomers, including butyl acrylate, (meth)acrylates, (meth)acrylamides, acrylonitrile, styrene [30,31,32] and its derivatives, butadiene, vinyl acetate, N-vinylpyrrolidone [27], and vinyl pyridines [20,21,22,33], and at the same time, the kinetics of it can be simulated, as shown in several publications [9,10,11,34,35]. Due to these flexibilities, RAFT polymerization was selected as an optimal polymerization technique for the synthesis of PDMAPAm homopolymer in a previous publication [19].

The quest to comprehend the kinetics of the polymerization process and its impact on the characteristics of the final products has been integral to the evolution of the RAFT polymerization method since its invention [36,37]. Reaction kinetic models are a vital component in this area of research, as evidenced by numerous studies in the literature. So far, several studies have been conducted regarding the kinetic modeling of bulk and solution RAFT polymerization of styrene [35,38,39,40,41,42], methyl methacrylate (MMA) [42,43,44,45], dodecyl methacrylate [46], 4-vinyl pyridine [9,10,12], NIPAM [47,48], and DMAEMA [49,50,51]. In some of them, the model has been used as a tool for upscaling the polymerization process [9,11].

Therefore, this research demonstrates the development of a kinetic model, the application of the validated kinetic model in upscaling of the synthesis of the poly(N-[3-(dimethylamino)propyl]-acrylamide)-*b*-poly(methyl methacrylate) (PDMAPAm-*b*-PMMA) diblock copolymer, and also preliminary results regarding the CO_2_-responsiveness of the diblock copolymer. The model investigates the polymerization kinetics and predicts the key properties of the homopolymer (PDMAPAm), such as molar mass, dispersity, and livingness. These properties are crucial for chain extension with MMA to synthesize PDMAPAm-*b*-PMMA. Additionally, the kinetic model is applied to scale up the synthesis of CO_2_-responsive PDMAPAm-*b*-PMMA from a 10 mL lab flask to a 100 mL glass reactor, which can facilitate the fabrication of CO_2_ membrane adsorbers on a larger scale for CO_2_ capture.

## 2. Materials and Methods

### 2.1. Materials

N-[3-(Dimethylamino)propyl]acrylamide (stabilized with MEHQ) (DMAPAm, >98.0%, TSI, Zwijndrecht, Belgium) was first dissolved in 1,4 dioxane (≥99.8%, Merck, Darmstadt, Germany) and percolated through a column of basic aluminum oxide (>98%, Sigma-Aldrich) before use to remove the inhibitor. Then, 2,2′-Azobis(2-methylpropionitrile) (AIBN, 98%, Sigma-Aldrich, Taufkirchen, Germany, stored at 4 °C) and 4-cyano-4-[(dodecylsulfanylthiocarbonyl)-sufanyl]pentanoic acid (CDTPA, 97%, Sigma-Aldrich, Taufkirchen, Germany, stored at 4 °C) were added into the monomer and solvent mixture. Methyl methacrylate (99%, stabilized with ≤30 ppm MEHQ, Sigma-Aldrich, Steinheim, Germany) was also freshly percolated with the same procedure for the same reason. Nitrogen was purchased by Linde (Hamburg, Germany, 99.999% purity). The copolymer was precipitated in n-hexane (99%, Sigma-Aldrich, Taufkirchen, Germany). The proton nuclear magnetic resonance (^1^H NMR) measurements were carried out in deuterated chloroform-d1 (CDCl_3_, 99.8% containing 0.03% (*v*/*v*) TMS, Sigma-Aldrich, Taufkirchen, Germany, stored at 4 °C). The gel permeation chromatography (GPC) measurements were carried out with the use of N,N-dimethyl acetamide (DMAc, ≥99.9%, Sigma-Aldrich, Taufkirchen, Germany) as the solvent and eluent of the system.

#### 2.1.1. Synthesis of PDMAPAm via RAFT Polymerization

For the kinetic experiments of PDMAPAm synthesis via RAFT polymerization, CDTPA (36.3 mg, 0.09 mmol, 1 eq.), AIBN (14.93 mg, 0.09 mmol, 1 eq.), and percolated DMAPAm (2800 mg, 18.00 mmol, 200 eq.) were dissolved in 2.4 mL of 1,4-dioxane (20 wt% monomer content) and separately added into small 10 mL flasks. The mixture in the flasks was degassed with nitrogen in a cold-water bath at 15 °C for 20 min (see the detailed synthesis protocol in the Supplementary Materials, Table S1). Following this, polymerization was conducted in a 10 mL glass flask using a thermo-shaker set at 70 °C and 250 rpm for 4 h, and every half an hour, the reaction in one of the flasks was stopped. The reaction was terminated by ice-cooling (0 °C) and exposure to air. Samples for ^1^H NMR and GPC were taken to determine the monomer conversion and molecular weight of the synthesized polymer, respectively. The final product was precipitated in n-hexane, followed by filtration and drying in a vacuum oven at 35 °C and a reduced pressure of 1 mbar overnight.

For upscaling, the DMAPAm was polymerized in a 100 mL glass reactor by applying the same recipe as mentioned above. A total of 10 g of homopolymer was synthesized (see more in the Supplementary Materials, Table S2), which was used for the synthesis of the diblock copolymer PDMAPAm-*b*-PMMA.

#### 2.1.2. Synthesis of PDMAPAm-b-PMMA via RAFT Polymerization

To synthesize the PDMAPAm-*b*-PMMA diblock copolymer, dried PDMAPAm (2600 mg, 0.126 mmol, 1 eq.), which served as the macro-RAFT/macro-stabilizer; MMA (1000 mg, 10 mmol, 100 eq.); and AIBN (17 mg, 0.126 mmol, 1 eq.) were dissolved in 15 mL of 1,4-dioxane (refer to the detailed synthesis protocol in the Supplementary Materials, Table S3). The mixture was degassed with nitrogen in a cold-water bath at 15 °C for 20 min. Polymerization was then conducted in a 30 mL glass flask using a thermo-shaker at 70 °C and 250 rpm for 24 h. The reaction was terminated by ice-cooling (0 °C) and exposure to air. Samples for ^1^H NMR and GPC were taken to determine the MMA conversion and the molecular weight of the synthesized diblock copolymer, respectively. The polymer was precipitated in n-hexane and dried in a vacuum oven at 35 °C and 1 mbar for 24 h.

### 2.2. Analytics

#### 2.2.1. Proton Nuclear Magnetic Resonance Spectroscopy (^1^H NMR)

^1^H NMR spectroscopy experiments were performed using a Bruker AV500 spectrometer (Bruker, Rheinstetten, Germany) and a Spinsolve Carbon 80 benchtop spectrometer (80 MHz, Magritek GmbH, Aachen, Germany). The spectra obtained with the Bruker AV500 were recorded by applying a 10 ms 90° pulse at a sample temperature of 298 K. Sixteen scans were recorded with a relaxation delay of 3 s. In a similar way, the experiments with the Spinsolve Carbon 80 were accomplished using the proton+ protocol with 4 scans, an acquisition time of 6.4 s, a repetition time of 1 min, and a pulse angle of 90°. The sample concentrations were 20 g L^−1^ and CDCl_3_. The ^1^H NMR spectra were analyzed with the software MestReNova 10.0 (Mestrelab Research, Santiago de Compostela, Spain). 1,3,6-trioxane was used as an internal standard. DMAPAm conversion from macro-RAFT synthesis was estimated by comparing the integration of double-bond proton areas in the sample before and after the reaction, which was around 90%. Using the same method, the conversion of methyl methacrylate in the second block synthesis, also measured in CDCl_3_, was calculated from the decrease in the integral of the monomer peaks, which was also approximately 90%.

#### 2.2.2. Gel Permeation Chromatography (GPC)

The apparent molecular weight distributions of the PDMAPAm homopolymers (macro-RAFT agents) and PDMAPAm-*b*-PMMA diblock copolymers were measured via GPC at 50 °C with DMAc with the addition of lithium chloride (0.1 M) as an eluent. A Waters 717 plus instrument (Waters, Milford, MA, USA) equipped with PSS GRAM columns (PSS GmbH, Mainz, Germany) [GRAM pre-column (dimensions 8–50 nm) and two GRAM columns of different porosity (3000 and 1000)] with dimensions of 8 × 300 mm and a particle size of 10 µm was used. The samples were measured at a flow rate of 1 mL min^−1^ using a VWR-Hitachi 2130 pump (VWR Hitachi, Darmstadt, Germany) and a VWR-Hitachi L2490 RI (refractive index) detector (VWR Hitachi, Darmstadt, Germany). The GPC equipment was calibrated with narrow PMMA standards, and the data were analyzed using PSS WinGPC UniChrom software, version 1.0, Agilent Technologies, Inc. 2023 (PSS GmbH, Mainz, Germany).

## 3. Results

### 3.1. Preliminary Remarks on Kinetic Experiments and Polymer Synthesis

**Figure 1:**
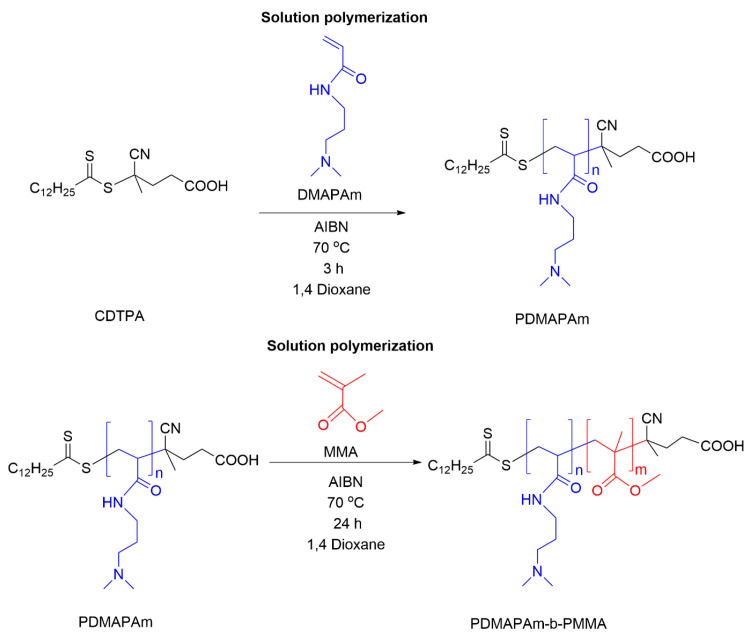
Synthesis of PDMAPAm-*b*-PMMA using PDMAPAm as macro-RAFT agent.

As described in previous work [19], the synthesis of PDMAPAm-*b*-PMMA involved two-step polymerization of the two monomers, as shown in Figure 1. First, poly(N-[3-(dimethylamino)propyl]acrylamide) (PDMAPAm) was synthesized at 70 °C by RAFT solution polymerization of N-[3-(dimethylamino)propyl]acrylamide in 1,4-dioxane. Subsequently, PDMAPAm served as a macroRAFT agent for the synthesis of a PDMAPAm-*b*-PMMA diblock copolymer by extending the chain via RAFT solution polymerization.

The aim was to synthesize a PDMAPAm-*b*-PMMA diblock copolymer with ${\overset{-}{M}}_{w,\mathrm{app}}=40\;\mathrm{kDa}$ and a 70:30 = PDMAPAm/PMMA ratio in order to have a large amount of PDMAPAm, which is a functional material/block that shows CO_2_ responsivity. To achieve this goal, it was very important to develop a kinetic model of polymerization that allows the study of the kinetics of polymerization and optimization of the synthesis protocol in order to obtain a polymer with the desired polymer properties, obtaining a great advantage of digitalized synthesis and reducing possible experimental attempts.

### 3.2. Mark–Houwink–Sakurada Parameters

The GPC evaluation of PDMAPAm and PDMAPAm-*b*-PMMA is performed with the PMMA standard, which is not a 100% fit to the molecular structure of the PDMAPAm-based copolymer. Therefore, the Mark–Houwink parameters of PDMAPAm are required to determine the actual apparent molar mass of the synthesized polymer. Using a similar procedure to that described in our previous publication [9], the Mark–Houwink parameters of PDMAPAm are determined by measuring the intrinsic viscosity (see Supplementary Information, Figures S1 and S2) of PDMAPAm with different molar masses dissolved in THF (Table 1).

The Mark–Houwink parameters a and *K* vary depending on the specific polymer–solvent system. For solvents, an a-value of 0.5 indicates a theta solvent, while an a-value of 0.8 is characteristic of good solvents [52,53]. It is evident from the results that the Mark–Houwink parameters of PDMAPAm are also in this range and agree well with the Mark–Houwink parameters of other N-containing polymers [54,55,56]. For PDEAEMA, PDMAEMA, PDMAPMAE [54], PNEAEMA, and PMOMA [56], the value of a changes between 6.00 × 10^−1^ and 7.5 × 10^−1^, and the a-value of PDMAPAm also falls within this range.

As for the K value of the other N-containing polymers, it ranges from 2.10 × 10^−4^ to 0.5 × 10^−4^ $\frac{dL}{g}$, whereas the figure is equal to 1.29 × 10^−4^ for our homopolymer. By knowing the Mark–Houwink parameters of PDMAPAm and PMMA [57,58] determined by viscosimetry measurement, the real ${\overset{-}{M}}_{w}$ of PDMAPAm is calculated using Equation (1) [59].

(1) $${{\overset{-}{M}}_{w},}_{PDMAPAm}={\left[\frac{K_{PMMA}}{K_{PDMAPAm}}\right]}^{\left(\frac{1}{a_{PDMAPAm}+1}\right)}\cdot {\left[{{\overset{-}{M}}_{w},}_{PMMA}\right]}^{\left(\frac{a_{PMMA}+1}{a_{PDMAPAm}+1}\right)}$$

**Figure 2:**
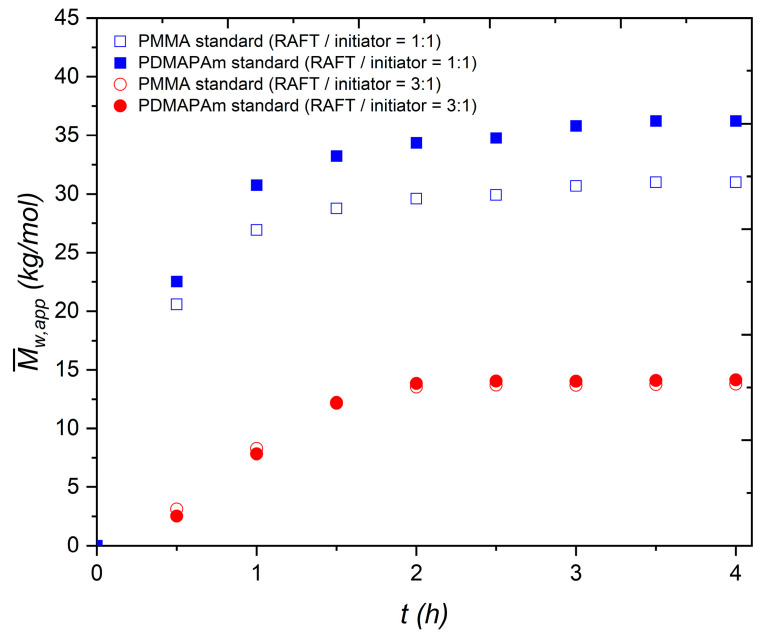
Correction of GPC evaluation. Evolution of weight-average molar masses of PDMAPAm from RAFT/initiator = 1:1 and RAFT/initiator = 3:1 experiments evaluated with PMMA and PDMAPAm standards.

In Figure 2, the weight-average molecular weights of PDMAPAm determined using the Mark–Houwink parameters of PMMA, with those determined using the estimated Mark–Houwink parameters of PDMAPAm, are presented. It can be seen from the diagrams that the evaluations with PMMA and PDMAPAm standards generally provide similar results for molecular weights below 20 kDa. The molar masses evaluated against the estimated PMMA parameters overlap with the molar masses evaluated against the estimated PDMAPAm parameters at higher RAFT-to-initiator ratios, but a slight discrepancy is observed as the ratio decreases. This occurs because at higher molecular masses than 20 kDa, the difference between the weight-average molecular masses determined using PMMA and PDMAPAm standards increases due to the different Mark–Houwink parameters of PDMAPAm and PMMA in THF.

### 3.3. Model Description

#### 3.3.1. Structure of Model

**Scheme 1:**
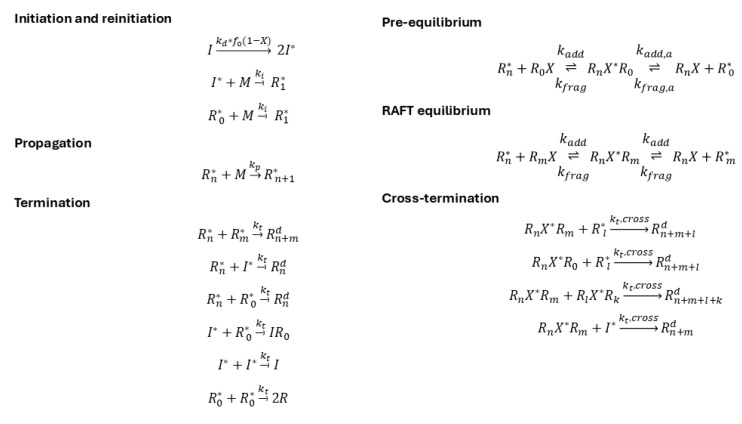
Reaction steps of kinetic model. *I*, M, $R_{n}^{d}$, $R_{0}X$, and $R_{n}X$ are initiator, monomer, terminated polymer, RAFT agent, and RAFT intermediate with one chain length, respectively. $I^{*}$, $R_{0}^{*}$, $R_{i}^{*}$, $R_{n}{X^{*}R}_{0}$, and $R_{n}{X^{*}R}_{m}$ are initiator radical, small leaving group left from RAFT agent, growing radical, RAFT intermediate radical with one chain length, and RAFT intermediate radical with two chain lengths.

The model developed by De Rybel et al. [41] and F. Kandelhard et al. [9] for the RAFT polymerization of styrene and 4-vinyl pyridine was selected as the base for this study due to its comprehensive consideration of all critical reaction steps, which are initiation, re-initiation, propagation, termination by recombination, cross-termination, pre-equilibrium, and RAFT equilibrium reactions (Scheme 1). Since the monomer concentration in solvent is 20% *w*/*w*, the gel effect for the termination reaction was not considered. There were two approaches to cross-termination in the literature. The first one is proposed by Barner-Kowollik et al. [23], who posit that the intermediate radical generated during the RAFT polymerization process is relatively stable and has a long lifespan. This model is referred to as the slow fragmentation model, which suggests negligible cross-termination of the intermediate radical and a high equilibrium constant (approximately $k_{add}/k_{frag}$ = 10^4^ to 10^8^) for the reaction between a growing radical and a dormant/RAFT-intermediated radical. The second one, which is proposed by Monteiro et al. [60], suggests that there is considerable cross-termination of the intermediate radical with other free radicals in the solution. This model is known as the intermediate radical termination model, predicting equilibrium constants that are several orders of magnitude lower than those of the slow fragmentation model (around a maximum of 10^2^). In this study, the model of Monteiro et al. [60] is considered because it is assumed that $k_{add}/k_{frag}$ will not be higher than 10^2^ and cross-termination of radical RAFT species with two chain lengths ($R_{n}{X^{*}R}_{m}$) is possible with an oligomeric growing radical ($R_{i})$ ($i_{c}=2\;\&\;\alpha_{c}=0.33)$ [61], as shown in Equations (2) and (3). Additionally, $R_{n}{X^{*}R}_{0}$ can also cross-terminate with primary radicals and growing radicals, as assumed in the previous publications [9,41].

(2) $$k_{t_{cross,i}}=k_{t_{cross,1}}{\left(i+1\right)}^{-\alpha_{c}}\;\;\;if\;i\leq i_{c}$$

(3) $$k_{t_{cross,i}}=0\;\;\;if\;i>i_{c}$$

#### 3.3.2. Reaction Rates

The rate constants for the reactions incorporated within the model are represented in Table 2. Since the initiator, AIBN, is used in this experiment, the $k_{d}$ and f values are taken from our previous publication and $k_{i}={10\cdot k}_{p}$ is assumed again [9]. In addition, the rate coefficients for the pre-equilibrium reaction steps are also added from a previous publication [9]. The kinetics of DMAPAm have not yet been studied, and therefore, there are no data on the rate coefficients of propagation and termination during the recombination of this monomer. However, DMAPAm can be considered a member of the family of N-containing methacrylates, similar to other monomers described in one of the recent publications of K. B. Kockler et al. [54]. This means that the propagation rate coefficient of DMAPAm should also be in the range described in this publication, which is assumed to be 2.50 × 10^3^ [L mol^−1^ s^−1^] for now, and this assumption will be checked at the end by comparing the rate coefficient of termination, estimated by applying parameter estimation, with the termination rate coefficients of other N-containing methacrylates belonging to the same family [54].

#### 3.3.3. Model Validation

The kinetic model was developed using Predici, version 11. Laplace. 1.5, 2023, a software tool designed for the numerical simulation of polymerization reactions, describing the critical reaction steps mentioned in the previous section.

**Figure 3:**
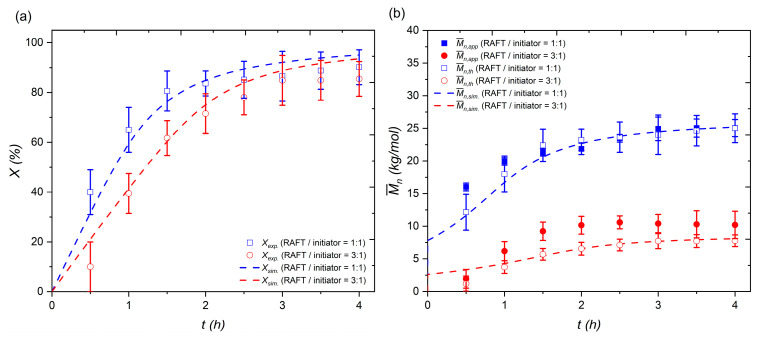
(**a**) Evolution of simulated (*X_sim._*) and experimental (*X_exp._*) DMAPAm conversion for two different RAFT/initiator ratios of 1:1 and 3:1. (**b**) Evolution of simulated (${\overset{-}{M}}_{n,\mathrm{sim}.}$), apparent (${\overset{-}{M}}_{n,\mathrm{app}}$), and theoretical (${\overset{-}{M}}_{n,\mathrm{th}}$) number-average molar masses within period of 4 h for two different RAFT/initiator ratios of 1:1 and 3:1.

Altogether, two reaction kinetic experiments were performed with two different RAFT/initiator ratios (CDTPA/AIBN = 1:1 and 3:1). The reaction rate coefficients of cross-termination, termination by recombination, and addition–fragmentation reactions were estimated by fitting the kinetic model to the experimental data of monomer conversion (*X_exp._* %), the theoretical number-average molar mass (${\overset{-}{M}}_{n,\mathrm{th}}$, which was estimated using the equation existing in the literature [7,20,21] based on monomer conversion determined with ^1^H-NMR measurement), and the apparent weight-average molar mass (${\overset{-}{M}}_{w,\mathrm{app}}$, measured with GPC). For this fitting, a parameter estimation mode in the software Predici was used. In the parameter estimation, the data from the two experiments were inserted into the model, and the data sets of the experiments were fitted together, as shown in Figure 3.

As depicted in Figure 3, the model accurately predicts the evolution of conversion and average molar mass. In particular, Figure 3a represents a complete overlap between the experimental (*X_exp._*) and simulated monomer conversion (*X_sim._*) data, whereas Figure 3b demonstrates that the experimental number-average molar mass (${\overset{-}{M}}_{n,\exp.})$ data align closely with the simulated (${\overset{-}{M}}_{n,\mathrm{sim}.}$) data throughout the entire reaction time. There is a minimal deviation of the apparent number-average molecular weight not only from the simulated ${\overset{-}{M}}_{n}$ but also from the theoretical ${\overset{-}{M}}_{n}$, which could be due to the fact that the used PMMA standard in this study is only valid at ${\overset{-}{M}}_{w,\mathrm{app}}$ < 20 kDa, as described in the literature [57], and this PMMA standard was chosen for comparison in our study because these Mark–Houwink parameters of PMMA were also determined by viscosimetry [57]. Therefore, better overlapping of ${\overset{-}{M}}_{n,\mathrm{app}}$ with ${\overset{-}{M}}_{n,\mathrm{th}}$ can be seen at a higher molar mass than this, especially in the case of RAFT/initiator = 1:1. In addition to this, at the beginning of the reaction, based on the simulation result, the ${\overset{-}{M}}_{n}\;$value in both cases increases very fast, which may be related to the fast decomposition of AIBN at 70 °C, which leads to the formation of a high amount of initiator radical at the start, as described in previous publications [11,62,63]. In addition, after 3 h of reaction time, the monomer conversion and the number-average molecular weight increase at a negligibly slow rate, which means the reaction can be terminated earlier than after 24 h [19].

From the fitting mentioned before, the values of rate coefficients of cross-termination, termination by recombination, and addition-–fragmentation reactions were estimated (Table 3).

The estimated rate coefficients agree quite well with the values available in the literature [9,35,41,60]. The assumption made regarding the possible cross-termination of the intermediate RAFT radical (which possesses two chain lengths) with the oligomeric growing radical was proven to be correct, as the equilibrium constant for the RAFT equilibrium ($K=\frac{k_{add}}{k_{frag}}$) was calculated to be around 5, which is lower than 10^2^. This means that this model follows the intermediate radical termination model of Monteiro et al. [60], predicting equilibrium constants that are several orders of magnitude lower than those of the slow fragmentation model of Barner-Kowollik et al. [23]. In other words, some of the radical RAFT species are consumed for a cross-termination reaction with oligomeric growing radicals [61], leading to an equilibrium constant several orders of magnitude lower than those of the slow fragmentation model [23].

Another assumption was concerning the value of the propagation rate coefficient of DMAPAm, which was assumed to be 2.5 × 10^3^ [L mol^−1^ s^−1^], which is in the range of propagation rate coefficients of N-containing methacrylates. The termination rate coefficient of DMAPAm was estimated to be 4.00 × 10^6^ [L mol^−1^ s^−1^], which is the same as the termination rate coefficient of DMAEMA [51], which also belongs to N-containing methacrylates. This means that the assumption regarding the kp value was correct, since the final estimated value of k_t_ is also within the values of the termination rate coefficients of other N-containing methacrylates.

**Figure 4:**
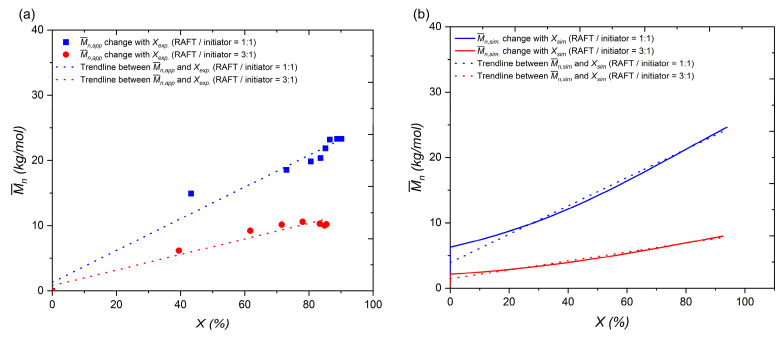
Change in number-average molar mass with monomer conversion. (**a**) Apparent number-average molar mass (${\overset{-}{M}}_{n,\mathrm{app}}$) (from GPC) change with monomer conversion (*X_exp._*) (from proton NMR) for both RAFT/initiator ratios of 1:1 and 3:1 and their corresponding linear trendlines. (**b**) Simulated number-average molar mass (${\overset{-}{M}}_{n,\mathrm{sim}.}$) change with simulated monomer conversion (*X_sim._*) for both RAFT/initiator ratios of 1:1 and 3:1 and their corresponding linear trendlines.

To investigate the kinetics of the living reaction, a change in the number-average molar mass (${\overset{-}{M}}_{n}$) of the polymer as a function of the monomer conversion (*X*%) is observed. Figure 4a indicates this change based on the experimental number-average molar mass (${\overset{-}{M}}_{n,\mathrm{app}}$ ) (from GPC) and the monomer conversion (*X_exp_*.) (from ^1^H NMR), whereas Figure 4b demonstrates this change using the simulated number-average molar mass (${\overset{-}{M}}_{n,\mathrm{sim}.}$) and monomer conversion (*X_sim_*.). It can be clearly seen that both the experimental and simulated data show that ${\overset{-}{M}}_{n}$ changes linearly with the monomer conversion, which means that the system exhibits pseudo-living reaction kinetics [11,64,65]. This linear increase is due to the controlled nature of RAFT polymerization, where the chain transfer agents regulate the growth of polymer chains, ensuring that all chains grow simultaneously and at a similar or uniform rate [66,67,68], which, in the end, makes the molecular weight distribution remain narrow [64,65].

### 3.4. Model Prediction and Application for Scaling Up

**Figure 5:**
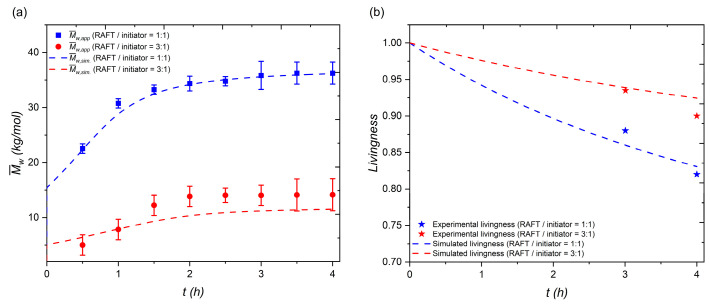
(**a**) Evolution of simulated (${\overset{-}{M}}_{w,\mathrm{sim}})$ and experimental (${\overset{-}{M}}_{w,\mathrm{app}}$) weight-average molar masses for two different RAFT/initiator ratios of 1:1 and 3:1. (**b**) Evolution of simulated livingness and experimentally determined livingness (based on ratio GPC peaks) for two different RAFT/initiator ratios of 1:1 and 3:1.

Using the validated model, the apparent weight-average molecular weight and the livingness of the PDMAPAm homopolymer were predicted and compared with the experimentally determined ${\overset{-}{M}}_{n,\mathrm{app}}$ values and the estimated homopolymer livingness from the experimental results. The simulation of the homopolymer livingness in the model is based on the equation existing in the literature [69]. In contrast, the experimental livingness of the PDMAPAm homopolymer could be determined either by comparing the theoretical ${\overset{-}{M}}_{n}$ of the diblock copolymer with the apparent ${\overset{-}{M}}_{n}$ or by analyzing the GPC raw data of the diblock copolymer synthesized from the macroRAFT of both kinetic experiments, as described in a previous publication [4]. Since the GPC data of diblock copolymers did not show any second peaks corresponding to any potential macroRAFT rest, the experimental livingness was estimated only by comparing the theoretical ${\overset{-}{M}}_{n}$ of the diblock copolymer with the apparent ${\overset{-}{M}}_{n}$ (see more in Supplementary Materials, Equation (S1)). The results of the comparison are shown in Figure 5.

It can be seen from Figure 5 that, in general, the apparent weight-average molecular weight (Figure 5a) and the livingness of the homopolymer (Figure 5b) within the reactions were predicted correctly for both kinetic experiments.

On closer inspection, there is a slight deviation in the simulated ${\overset{-}{M}}_{w}$ from the experimental values in the kinetic experiment with a higher RAFT agent concentration, where the maximum ${\overset{-}{M}}_{w}$ is close to 15 kDa, which is still outside the PMMA calibration range of GPC. The deviation is almost the same as in the ${\overset{-}{M}}_{n}$ comparison, which means that this could be related to the chosen PMMA standard (valid above 20 kDa) for the evaluation.

Furthermore, the model predicts the livingness, in other words, the chain-end fidelity of PDMAPAm, very well. It was determined that after 4 h of reaction, for a RAFT/initiator ratio of 3:1, the experimental livingness is around 90%, whereas the value reaches at 83% for the lower-RAFT-concentration case, as illustrated in Figure 5; these values are equivalent to 4–5 DMAPAm monomeric units, which could be difficult to identify with GPC. These values of experimentally determined livingness agree quite well with the predicted livingness of the PDMAPAm from both kinetic experiments. There is a slight deviation in the predicted livingness for the lower-RAFT-concentration experiment, but the deviation is so small that it can be considered negligible.

Moreover, based on the model prediction, the livingness of the PDMAPAm homopolymer decreases with reaction time for both RAFT/initiator ratios, as shown in Figure 5b, because more radicals undergo termination during the polymerization, as stated in previous publications [9,10]. As for comparing the reaction kinetic experiments with each other, the simulation shows that a higher RAFT concentration leads to higher livingness of the homopolymer in the end. This trend is also followed by the experimental livingness of the synthesized PDMAPAm. Additionally, after 3 h of reaction time, no substantial change is observed in the value of the average molar masses. However, the livingness of the homopolymer still decreases with the same gradient. At the same time, experimentally, it can be seen that macroRAFT livingness is higher at the end of 3 h than at the end of 4 h of reaction time. Therefore, the same average molar mass is obtained by stopping the reaction earlier with even higher livingness, as experimentally proven in a previous publication [19].

**Figure 6:**
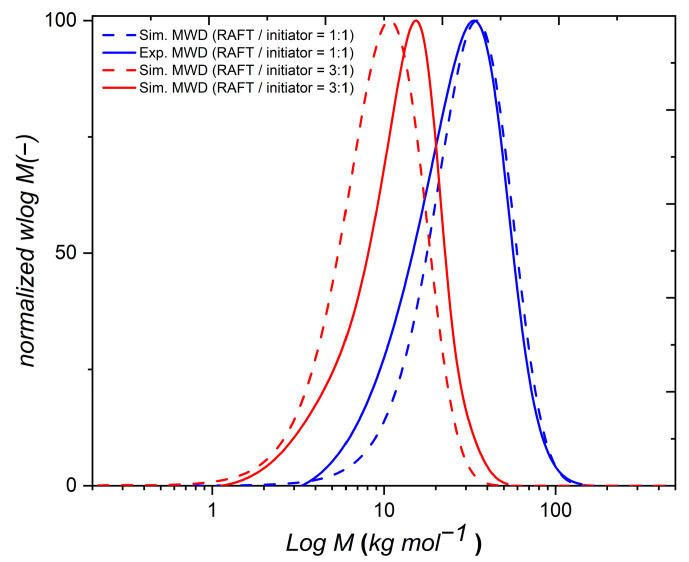
Molecular weight distributions (MWDs) of PDMAPAm. Simulated (Sim.) MWD represented by dashed lines and experimental (Exp.) MWD shown as continuous lines for both RAFT/initiator ratios of 1:1 and 3:1 at end of 3 h reaction time.

In Figure 6, the simulated molecular weight distributions (MWDs) are correlated with the experimental MWDs of the polymerization product measured with GPC for both experiments after 3 h of reaction. It can be clearly seen from the data that the model-predicted dispersity for both reactions is well estimated, because the breadth of the simulated and experimental MWDs for both reactions is the same as that calculated by the model. However, generally, the experimental MWDs and MWDs simulated by the model do not overlap for the experimental RAFT/initiator ratio of 3:1, and the peak point, M_p_ (representing the most probable molecular weight of the polymer chains in the sample), of the experimental and simulated MWDs are not close to each other. As mentioned before, this discrepancy was observed between simulated ${\overset{-}{M}}_{n}$ and experimental ${\overset{-}{M}}_{n}$, and this was justified with the evaluation standards of the GPC results.

Considering the predicted ${\overset{-}{M}}_{w}$, livingness, and optimal reaction time for both the kinetic experiment protocols and comparing the prediction results of the kinetic model with our aim stated at the start of this study, the synthesis protocol with a RAFT/initiator ratio of 1:1 is considered to be optimal, since it leads to more-or-less acceptable livingness, which leads to the desired weight-average molar mass for the homopolymer (25 kDa) at the end of the 3 h of reaction time and, at the same time, a PDMAPAm/PMMA block ratio of 70:30.

Using the optimal recipe (RAFT/initiator ratio of 1:1), the synthesis of the homopolymer PDMAPAm was increased from 10 mL lab-scale synthesis to 100 mL glass reactor synthesis. Afterwards, PDMAPAm was used as a macroRAFT to synthesize a PDMAPAm-*b*-PMMA diblock copolymer with the desired molar mass and PDMAPAm/PMMA block ratio. Based on the ^1^H NMR result, the conversion of MMA for the synthesis of the second block was calculated to be over 90%.

**Figure 7:**
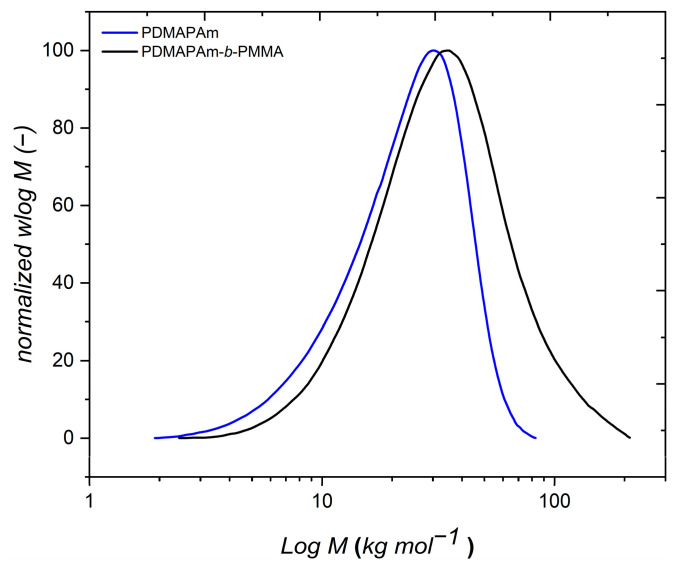
Molar mass distribution curve of PDMAPAm and PDMAPAm-*b*-PMMA measured with GPC.

In Figure 7, the MWDs of the synthesized PDMAPAm and PDMAPAm-*b*-PMMA in the glass reactor are presented as measured with GPC. Comparing the MWDs of both the homopolymer and diblock copolymer, it is obvious from the graph that the MWD of PDMAPAm shifts slightly to higher molar masses as its chain is extended with the second monomer MMA. This shift clearly shows the growth of the second block [7,9,10,19].

Moreover, Table 4 shows that the weight-average molar mass of the synthesized PDMAPAm-*b*-PMMA is around 40 kDa, and the ratio of the PDMAPAm block to the PMMA block is around 70:30, in line with the aim stated at the beginning. This means that the targeted molecular characteristics for the diblock polymer were achieved.

### 3.5. Preliminary Results of CO_2_-Responsiveness of Polymer

To demonstrate the preliminary results of the CO_2_-responsive behavior of the PDMAPAm-*b*-PMMA diblock copolymer, a small experiment was performed.

First, the pH of commercial water by “Viva con Agua” (Hamburg, Germany) was measured to be around 8.25, and then, by degassing, it was saturated with CO_2_, which reduced the pH to 5.29 due to the formation of carbonic acid ($\;{CO}_{2\;}+H_{2}O\underset{\leftarrow }{\to }H_{2}CO_{3}$), which dissociates into bicarbonate ($\;{HCO_{3}}^{-}$) and hydrogen ions ($H^{+}$) [70], lowering the pH.

**Figure 8:**
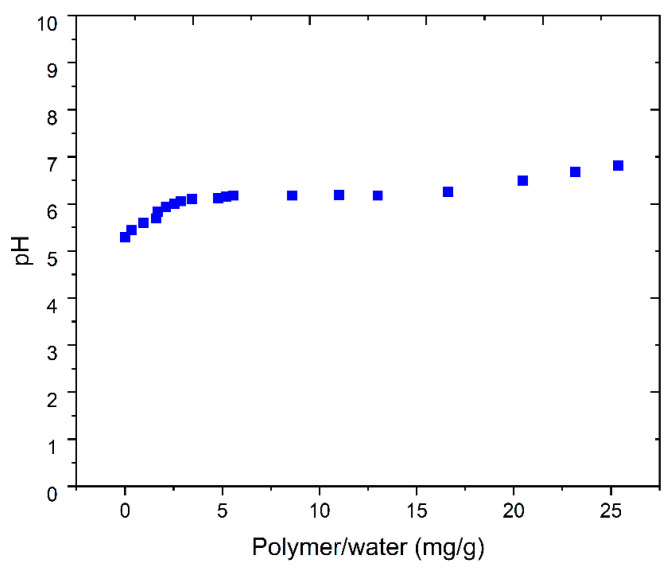
Resultant pH changes in CO_2_-saturated water with dissolved diblock copolymer.

The diblock copolymer was incrementally introduced into the CO_2_-saturated water, and the resultant pH changes in the solution were systematically measured at room temperature (23 °C) (Figure 8).

It can be clearly seen from Figure 8 that as the PDMAPAm-*b*-PMMA diblock copolymer is added into the CO_2_-saturated water, its pH increases from 5.29 to 7.10. This might be related to the fact that as soon as the diblock copolymer is added into the CO_2_-saturated water, the tertiary amine group on the PDMAPAm block can theoretically react with CO_2_ and water (${R_{3}N+CO}_{2\;}+H_{2}O\underset{\leftarrow }{\to }\;R_{3}NH^{+}+{HCO_{3}}^{-})$ [71,72,73] and form bicarbonate ions. As a result, this can reduce the proton concentration in the water and results in an increase in the pH value. This interaction of the tertiary amine group with water and CO_2_ and the formation of bicarbonate ions show that the diblock copolymer is CO_2_-reponsive, as mentioned in several publications [71,72], and could be a potential material for CO_2_ capture [71,72].

## 4. Conclusions

A reaction kinetic model for the RAFT polymerization of DMAPAm was developed and validated with two different reaction kinetic experiments. Good agreement was obtained between simulated and experimental monomer conversion and the number-average molar masses. To validate the developed model along with more concrete parameter estimation, the Mark–Houwink parameters of PDMAPAm were estimated in order to adapt the molar masses of the polymer. Within the validation, it was determined that propagation and termination by the recombination rate coefficients of DMAPAm follow the values of the corresponding rate coefficients of other N-containing monomers. At the same time, it was identified that the Mark–Houwink parameters of PDMAPAm fall within the range of the parameters of N-containing methacrylate polymers. This means that DMAPAm also belongs to the family of N-containing monomers, and the developed model is applicable to the RAFT polymerization of other N-containing monomers. In addition, both simulated and experimental ${\overset{-}{M}}_{n}$ as a function of the monomer conversion relation showed that the system has pseudo-living reaction kinetics, referring to the existence of the RAFT kinetics of the system. After the validation, the model was used to predict livingness, in other words, the chain-end group fidelity; weight-average molar mass; and MWD of the homopolymer. Using the kinetic model, the synthesis protocol of the homopolymer PDMAPAm was optimized to achieve the desired ${\overset{-}{M}}_{w}$ and livingness with the highest monomer conversion in an optimal reaction time. In the end, the optimal recipe was implemented to synthesize the CO_2_-responsive diblock copolymer of PDMAPAm-b-PMMA with the desired molar mass (40 kDa) and molar mass ratio of the PDMAPAm block to the PMMA block (70:30) on a larger scale, which can be implemented in direct air capture technology.

In the future, to make the prediction of the simulation more precise and the examination of the reaction kinetics more thorough, a calorimeter can be utilized to determine the polymerization rate experimentally, in other words, to determine the exact propagation rate coefficient of the polymerization, and to investigate the energy balance of the RAFT polymerization of DMAPAm, which could also open a door to optimizing the reaction in terms of energy consumption and to bring the process to industry.

## Figures and Tables

**Table 1:** Determined Mark–Houwink–Sakurada parameters of PDMAPAm in THF.

$K_{PDMAPAm}(\frac{dL}{g})$	$a_{PDMAPAm}$
1.29 × 10^−4^	6.25 × 10^−1^

**Table 2:** Rate coefficients used for reaction kinetic modeling.

Rate Coefficient	A [Variable Unit]	$E_{A}$ [K]	k (at 70 °C) [Variable Unit]	Reference
$k_{d}$	1.58 × 10^15^ [s^−1^]	1.55 × 10^4^	3.64 × 10^−5^ [s^−1^]	[9]
$f_{0}$	-	-	0.6	[9]
$k_{p}$	2.44 × 10^6^ [L mol^−1^ s^−1^]	2.36 × 10^3^	2.5 × 10^3^ [L mol^−1^ s^−1^]	[54]
$k_{i}$	$k_{i}={10\cdot k}_{p}$			[9]
$k_{t,cross}$	Estimated in this work			
$k_{t}$				
$k_{add}$				
$k_{\mathrm{frag}}$				
$k_{add,a}$	-	-	1.00 × 10^4^ [s^−1^]	[9]
$k_{\mathrm{frag},a}$	-	-	2.00 × 10^4^ [L mol^−1^ s^−1^]	[9]

**Table 3:** Estimated values of rate coefficients of cross-termination, termination by recombination, and addition–fragmentation reactions from parameter estimation (PE).

$k_{t,cross}$	$k_{t}$	$k_{add}$
8.00 × 10^9^ [L mol^–1^ s^–1^]	4.00 × 10^6^ [L mol^−1^ s^−1^]	1.00 × 10^4^ [L mol^−1^ s^−1^]

**Table 4:** The apparent number and weight-average molecular weight along with the dispersity of the synthesized PDMAPAm-*b*-PMMA.

Polymer	${\overset{-}{M}}_{n,\mathrm{app}}(kDa)$	${\overset{-}{M}}_{w,\mathrm{app}}(kDa)$	Ɖ
PDMAPAm_165_	19	26	1.4
PDMAPAm_163_-*b*-PMMA_130_	26	39	1.5

## Data Availability

The characterization data are available upon request from the authors.

## Supplementary Materials

The following supporting information can be downloaded at: https://www.mdpi.com/article/10.3390/polym17081115/s1, Table S1. Recipe of kinetic experiment for PDMAPAm synthesis; Table S2. Recipe for scale-scaling up of PDMAPAm synthesis; Table S3. Recipe of PDMAPAm-b-PMMA synthesis; Figure S1. Reduced viscosity change with polymer concentration in THF for PDMAPAm homopolymers with different molar masses (15 kDa, 25 kDa, 42 kDa, 94 kDa); Figure S2. Plot of intrinsic viscosity versus molar mass of both PMMA and PDMAPAm homopolymers [57,58].

## Abbreviations

The following abbreviations are used in this manuscript:

DMAPAm	N-[3-(dimethylamino)propyl]-acrylamide
MMA	Methyl methacrylate
PDMAPAm-*b*-PMMA	Poly(N-[3-(dimethylamino)propyl]-acrylamide)-b-poly(methyl methacrylate)
RAFT	Reversible Addition−Fragmentation Chain-Transfer
CDTPA	4-cyano-4-[(dodecylsulfanylthiocarbonyl)-sufanyl]pentanoic acid
AIBN	2,2′-Azobis(2-methylpropionitrile)
CDCl_3_	Deuterated chloroform
THF	Tetrahydrofuran
4VP	4-Vinylpyridine
^1^H NMR	Proton Nuclear Magnetic Resonance Spectroscopy
GPC	Gel permeation chromatography
