# Supplementary material for: Optimizing the Synthesis of CO2-Responsive Polymers: A Kinetic Model Approach for Scaling Up
Source: Polymers (Basel). 2025 Apr 20;17(8):1115. doi: 10.3390/polym17081115 (PMC12031492; doi:10.3390/polym17081115)
Supplement: Supplementary file 1 [file polymers-17-01115-s001.zip › polymers-3576946-supplementary.pdf]

Recipes of experiments

Table S1. Recipe of kinetic experiment for PDMAPAm synthesis.

RAFT/initiator (eq.)	CDTPA (g)	AIBN (g)	DMAPAm (g)	1,4 Dioxane (g)	Total (g)
1:1	3.63E-02	14.93 E-03	2.80E+00	2.884	5.72E+00
3:1	1.22E-02	15 E-03	2.78E+00	2.871	5.66E+00

Table S2. Recipe for *scaling up* of PDMAPAm synthesis.

RAFT/initiator (eq.)	CDTPA (g)	AIBN (g)	DMAPAm (g)	1,4 Dioxane (g)	Total (g)
1:1	2.22E-01	7.51E-02	1.50E+01	6.03E+01	7.56E+01

Table S3. Recipe of PDMAPAm-*b*-PMMA synthesis.

PDMAPAm/AIBN/MMMA	MMA (g)	PDMAPAm (g)	AIBN (g)	1,4 Dioxane (g)	Total (g)
1:1:100	1.00E+00	2.60E+00	1.70E-02	1.55E+01	1.91E+01

Estimation of Mark–Houwink parameters

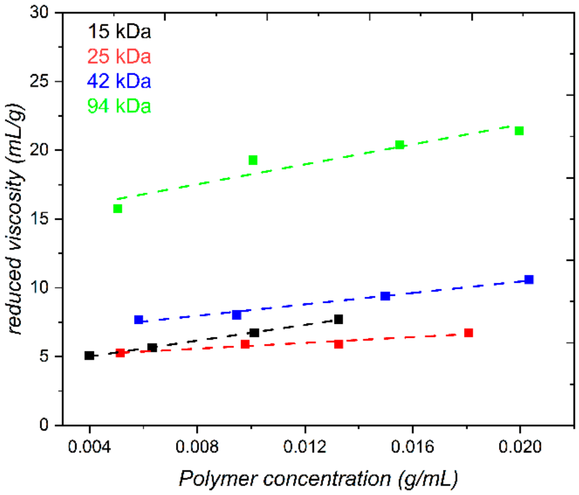

Figure S1. Reduced viscosity change with polymer concentration in THF for PDMAPAm homopolymers with different molar masses

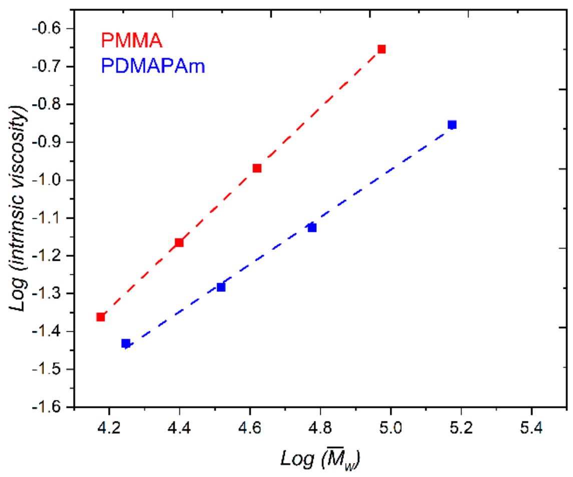

Figure S2. Plot of intrinsic viscosity versus molar mass of both PMMA and PDMAPAm homopolymers.

Generally, the intrinsic viscosity of the polymer solution is the point where the trendline describing reduced viscosity versus polymer concentration crosses the y-axis. These intrinsic viscosities were determined for the PDMAPAm polymers with different molar masses using Figure S1. As for the intrinsic viscosity and corresponding values of PMMA in THF, they were taken from the literature [1,2]. In the publication of J. Janca *et al.*, the Mark–Houwink parameters of PMMA were also determined using the viscosity measurement of the PMMA solution in THF in the same manner as we did for PDMAPAm.

Finally, the graph of the slope of $Log\ (intrinsic\ viscositity)$ ($\mu_{intinsic}$) versus $Log(\overline{M}_w\ )$ provides the *a*-value, whereas its cross-section refers to the *K*-value of the Mark–Houwink equation for PDMAPAm.

Determination of experimental livingness

$$Experimental\ livingness\ of\ PDMAPAm\ (\%) = 100 * (1 - \frac{\overline{M}_{n,th(PDMAPAm-b-PMMA)} - \overline{M}_{n,app(PDMAPAm-b-PMMA)}}{\overline{M}_{n,app\ (PDMAPAm)}}) \quad (S\ 1)$$

$\overline{M}_{n,th(PDMAPAm-b-PMMA)}$ —theoretical number-average molar mass of diblock copolymer based on MMA conversion determined using proton NMR.

$\overline{M}_{n,app(PDMAPAm-b-PMMA)}$ —apparent number-average molar mass of PDMAPAm-*b*-PMMA diblock copolymer measured with GPC.

$\overline{M}_{n,app\ (PDMAPAm)}$ —apparent number-average molar mass of corresponding PDMAPAm homopolymer (which was later exposed to chain extension with MMA) measured with GPC.

References

1. Wagner, H.L. The Mark–Houwink–Sakurada relation for poly (methyl methacrylate). *Journal of physical and chemical reference data* **1987**, *16*, 165-173.
2. Janča, J.; Vlček, P.; Trekoval, J.; Kolínský, M. Universal calibration in gel permeation chromatography for poly (methyl methacrylate) and poly (2-methoxyethyl methacrylate). *Journal of Polymer Science: Polymer Chemistry Edition* **1975**, *13*, 1471-1473.
